# Cadmium Minimization in Grains of Maize and Wheat Grown on Smelting-Impacted Land Ameliorated by Limestone

**DOI:** 10.3390/toxics12080532

**Published:** 2024-07-24

**Authors:** Fuqing Sui, Yanzheng Yang, Yong Wu, Jiali Yan, Haichao Fu, Chang Li, Shiyu Qin, Long Wang, Wenwen Zhang, Wei Gao, Hongen Liu, Peng Zhao

**Affiliations:** 1 Key Laboratory of Soil Pollution Control and Remediation of Henan Province, College of Resources and Environment, Henan Agricultural University, Zhengzhou 450046, China; fuqing.sui@henau.edu.cn (F.S.); 15516573717@163.com (Y.Y.); yong.wu@henau.edu.cn (Y.W.); haichaofu@henau.edu.cn (H.F.); changli@henau.edu.cn (C.L.); qinsy0418@henau.edu.cn (S.Q.); hnndwanglong@163.com (L.W.); zhangwenwen@henau.edu.cn (W.Z.); gaowei_1126@163.com (W.G.); liuhongen7178@126.com (H.L.); 2 College of Civil and Architecture Engineering, Chuzhou University, Chuzhou 239000, China

**Keywords:** Cadmium (Cd), limestone, acid soil, wheat, maize, accumulation

## Abstract

Cadmium (Cd) contamination in agricultural soils has emerged as a significant concern, particularly due to its potential impact on plant-based food. Soil pH reductions can exacerbate Cd mobility, leading to excessive accumulation in crops. While liming has been demonstrated as an effective method to mitigate Cd accumulation in rice grains in acid soils of southern China, its efficacy in remediating acid soils in northern China remains unclear. In this study, a multi-year field experiment was conducted on farmland impacted by zinc ore smelting at coordinates of 33.92° N 112.46° E to investigate the use of limestone for controlling Cd accumulation in wheat and maize grains. The results indicated that applying 7.5 t ha^−1^ of limestone significantly raised the soil pH from 4.5 to 6.8 as anticipated. Different rates of limestone application (2.25, 4.45, and 7.50 t ha^−1^) reduced Cd bioavailability in the soil by 20–54%, and Cd accumulation in wheat grains by 5–38% and maize grains by 21–63%, without yield penalty. The remediation effects were sustained for at least 27 months, highlighting limestone as a promising ameliorant for smelting-affected farmland in northern China.

## 1. Introduction

Cadmium (Cd) is a heavy metal that poses a significant health risk. Exposure to Cd can lead to kidney damage, osteoporosis, and itai-itai disease [1], resulting in its classification as a class one carcinogen. Over the past 30 years, rapid industrial development and a lack of regulations to prevent soil contamination have led to a concerning issue of soil Cd contamination [2]. According to the national soil pollution survey bulletin, 19.4% of farmland samples exceeded the national environmental standard, with 7% surpassing China’s national Cd threshold value, making Cd the top inorganic pollutant (http://www.mee.gov.cn/gkml/sthjbgw/qt/201404/t201404-17_270670.htm, accessed on 17 April 2014). Additionally, a study found that the Cd content in the soil plow layer in China is increasing at an average rate of 0.004 mg kg^−1^ yr^−1^ [3]. If this trend continues, it is projected that the average Cd content in the soil could double in just 50 years [2]. The soil Cd content of 0–23 cm in the famous long-term experimental site in Rothamsted, UK, increased from 0.17–0.51 mg kg^−1^ to 0.27–0.77 mg kg^−1^ from the 1850s to the 1980s, with an approximate average yearly increase of 0.7–2.7 μg kg^−1^ [4]. Similarly, surface soil Cd content increased 33% compared with the archived soil samples taken from nearly a century earlier in a long-term agronomic experimental field of Cornell University [5]. In New Zealand, soil total Cd levels are still increasing due to the phosphorus fertilization [6].

Elevated Cd content in soils can result from both natural processes and human activities. Natural processes, such as soil formation, are influenced by factors like parental materials, volcanic activity, and rock weathering. For example, in regions like Yunnan, Hunan, and Guangxi in southern China, the Cd concentration of arable land is notably high [7]. On the other hand, human activities, particularly during rapid industrialization in China, have significantly contributed to the increase in Cd levels in farmland [6,7]; These activities include air deposition from industrial and smelting processes, wastewater irrigation, as well as the use of manures, fertilizers, and pesticides [8,9].

Once in agricultural soils, Cd has been found to be taken up by plants more efficiently compared to many other metals and metalloids, such as chromium (Cr), lead (Pb), mercury (Hg), fluorine (F), gold (Au), titanium (Ti), silver (Ag), tin (Sn), zirconium (Zr), iron (Fe), and aluminum (Al), leading to concerns about food safety [10]. Cd transported to the aboveground tissues also varies among plant species, for example, maize, barley, oat, ryegrass, cocksfoot, and rice are often shoot Cd excluders [11]. It is estimated that approximately 90% of Cd intake in non-smoking human populations occurs through the food chain [12]. For a population living in a Cd-contaminated area, significantly higher Cd exposure and health risks were observed [13,14]. In Chinese populations, rice contributes to 65% and 38% of cadmium intake in southern and northern regions, respectively [15]. Therefore, reducing the entry of cadmium into the food chain is crucial for protecting human health. The Joint FAO/WHO Expert Committee on Food Additives (JECFA) set a limit of 0.4 mg kg^−1^ for polished rice, 0.2 mg kg^−1^ for wheat, and 0.1 mg kg^−1^ for maize (https://www.who.int/groups/joint-fao-who-expert-committee-on-food-additives-(jecfa), accessed on 10 July 2023). China has set a national limit of 0.2 mg kg^−1^ dry weight for rice (de-husked), and 0.1 mg kg^−1^ for wheat and maize. Several studies have shown that the cadmium content in rice often exceeds the national limit [16,17]. Geographical variations in cadmium content in polished rice grains have been reported in China [16]. A study on 471 high-yield rice cultivars in southern China revealed that the cadmium concentration in rice grains varied significantly, with some cultivars showing much lower cadmium accumulation than others [18]. While rice is known for its ability to uptake Cd, wheat is more efficient in translocating Cd to the aboveground tissues, potentially leading to excessive accumulation in grains [19]. This phenomenon was further supported by a meta-analysis examining the variation in grain Cd accumulation between wheat and rice [20]. Wheat accounts for 10.5% of human Cd intake in northern China due to its high consumption rates [15]. The capacity for Cd uptake varies significantly among wheat cultivars, with Cd concentrations in 132 cultivars from the Northern China Plain ranging from 1.0 to 34 mg kg^−1^ [21]. In terms of grain Cd concentration, all 25 wheat cultivars grown in a field affected by lead smelting in northern China exceeded the national limit of 0.1 mg kg^−1^ dry weight [22]. In another study, flour from wheat produced in the smelting area also exceeded the national limit and raised health concerns [23].

The accumulation of Cd in wheat grains is influenced by environmental factors, posing challenges for accurate phenotyping [24]. Soil properties, rather than total Cd content, play a significant role in the transfer of Cd into wheat grains [25]. The adsorption of Cd on solid phases of soil is highly dependent on pH [1,26]. The mobility and bioavailability of Cd increase as pH decreases [27]. Usually, a one-unit decrease in pH leads to a fourfold increase in Cd solubility [1,28]. Long-term tests have shown that 60–90% of Cd in soils can become activated at pH 4.0 [29]. What make matters worse is that significant acidification in croplands in China have been reported [30,31]. An investigation that included 1443 pairs of soil pH observations from co-located sites in 2008 and 2018 revealed that the soil pH in Henan province decreased by an average of 0.36 units, with more than 94% of croplands experiencing varying degrees of acidification [32]. One of the major sources of this acidification has been nitrogen fertilizer input [33]. Studies have shown a close relationship between the shift in topsoil pH and nitrogen input [32]. Soil acidification not only has negative impacts on the sustainability of agricultural systems but also increases the availability of Cd [2]. Studies revealed that pH is a powerful tool in the management of the Cd content of plants such as clover, lettuce, carrot ryegrass, and rice [34,35].

Liming of acid soils in southern China has been shown to effectively control rice grain cadmium (Cd) accumulation. However, the impact of liming in northern China on reducing Cd accumulation in wheat and maize grains has not yet been fully characterized. In the present study, we hypothesized that limestone addition could decrease Cd accumulation in wheat and maize grains in smelting-affected farmland in northern China. Here, we test the effect of limestone application on wheat and maize Cd accumulation in smelting-impacted land, intending to provide guidance for the management of Cd-contaminated soil.

## 2. Materials and Methods

### 2.1. Field Location

**Figure 1:**
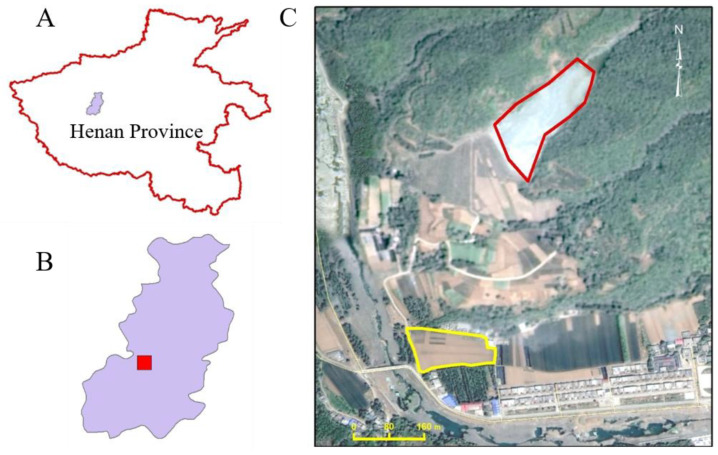
Field experiment location. (**A**) Location of Ruyang County (colored purple) in Henan Province; (**B**) map of Ruyang County (the experiment site location is painted in red); (**C**) details of the experimental site. The tailings were deposited (red outline) in a small valley on a slope. The experimental site (yellow outline) is located under the foot of the mountain (112.46° E 33.92° N).

Henan Province is in the northern part of China. A field experiment was set up in Ruyang County, Luoyang City, Henan Province, China (33.92° N 112.46° E) to investigate limestone addition on Cd-contamination remediation (Figure 1). Ruyang County experiences a warm, temperate continental, monsoon climate with average annual sunshine of 2177 h, a mean temperature of 14 °C, and mean rainfall of 690 mm. The experiment site was chosen based on preliminary investigation findings indicating soil contamination due to runoff and leaching from zinc-smelting tailings deposited in a nearby valley (Figure 1). It is important to note that the selected site is no longer receiving fresh contamination, thanks to a restoration project that altered the runoff patterns. Preliminary detailed investigation showed that the experimental site has a total Cd content of 0.63–0.99 mg kg^−1^ dry weight, a pH of 4.52–4.90, organic matter content of 8 g kg^−1^, and a loam texture with clay, silt, and sand content of 32%, 39%, and 29% respectively. Detailed properties of the tested soil can be found in Table S1.

### 2.2. Experimental Method

A model predicting the quantity of lime materials needed to increase the soil pH to the specific target was previously developed by Nanjing Agricultural University [10,36,37]. According to the model’s prediction, 7.5 t ha^−1^ of limestone was required to raise the soil pH from 5.0 to 6.5. In order to further investigate the soil pH’s response to the limestone addition, rates of 0, 2.25, 4.45, and 7.50 t ha^−1^ were applied in three independent replicates. Each plot had an area of 4 m × 4 m with 1 m of buffering areas between neighboring plots to prevent potential contamination, and followed randomized block design. Limestone (CaCO_3_ ≥ 98%, ≤2 mm) was broadcasted on the soil surface. The soil was plowed (20 cm) with a moldboard plow and then rotary hoed twice to ensure good mixing. Limestone was applied 2 weeks before maize sowing in 2019. Locally adapted maize cultivar Yudan185 and wheat cultivar Luomai9908 were used in the present study. Maize was sown on 15 May 2019, 10 May 2020, and 8 May 2021, and harvested on 28 September 2019, 26 September 2020, and 22 September 2021. Wheat was sown on 6 October 2019 and 8 October 2020, and harvested on 28 May 2020 and 30 May 2021.

### 2.3. Soil and Plant Sampling

Maize and wheat were sampled in three replicates, with each sample consisting of a mixture of three individual plants. Following the methodology outlined previously [38] (Sui. et al., 2019), the samples were processed by dividing them into shoots, roots, and grains, which were then ground into a fine powder for Cd determination. Soil samples were obtained by combining three cores from the 0–20 cm depth, which were air dried and sieved through a 2 mm sieve for analysis of soil pH and DTPA-Cd. The samples were collected at different stages: maize maturity stage in 2019 (3 months after treatment), wheat jointing stage in 2020 (9 months after treatment), maize maturity stage in 2020 (12 months after treatment), and wheat maturity in 2021 (27 months after treatment). Fertilization of maize and wheat followed local agricultural practices. A total of 750 kg ha^−1^ compound fertilizer (N-P_2_O_5_-K_2_O, 18-18-18) was used as a basal fertilizer before wheat sowing and 150 kg ha^−1^ urea was used as topdressing during the wheat jointing stage for the wheat season. For the maize season, 900 kg ha^−1^ compound fertilizer (N-P_2_O_5_-K_2_O, 14-24-7) was used. Wheat yield was calculated as the harvest for 1 m^2^ and multiplied by 10,000; maize yield was calculated as the harvest for 50 plants and multiplied by 675,000 plants ha^−1^.

### 2.4. Sample Analyses

Basic soil properties were determined as previously reported [36]. Soil pH was measured using a pH meter (FE28, Mettler Toledo, Zurich, Switzerland). Total metal concentrations in the soil were determined after digestion. Soil available Cd was extracted using 0.005 M DTPA according the national standard of China [39], which is an efficient extracting agent. Cd speciation in the soil was fractionated using the Tessier graded continuous extraction method, which includes water-soluble cadmium and the exchangeable form (EX-Cd), the carbonate-binding form (CB-Cd), the iron–manganese-oxide-binding form (OX-Cd), the organic-binding form (OC-Cd), and the residual form (RE-Cd) [40]. The Cd concentration in the extracts and digestions was analyzed using an atomic absorption spectrophotometer (PE900T, PerkinElmer, Norwalk, CT, USA). Standard soil samples GBW07427 and wheat flour samples GBW(E)100496 from the national standard material resource-sharing platform [41], as well as blanks, were used for quality control analysis. The recoveries of Cd in the standard materials ranged from 96% to 105%, indicating high quality in the sample analysis.

### 2.5. Data Collection and Statistics

SPSS 22.0 software was used for data analysis (IBM Corp., Armonk, NY, USA). The data were subjected to the Shapiro–Wilk analysis for the normality test and Bartlett analysis for equal variances test to check whether the ANOVA assumption were satisfied, followed by a one-way ANOVA. Data visualization was carried out by Sigma plot 14.0 (SYSTAT Corp., San Jose, CA, USA).

## 3. Results

### 3.1. Soil pH

**Figure 2:**
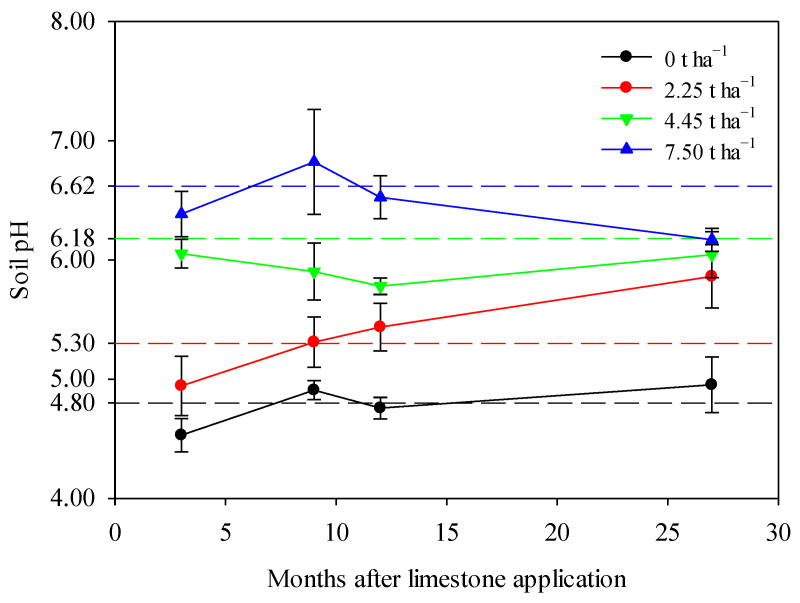
Limestone application significantly increased soil pH during a maize–wheat rotation in northern China after 3, 9, 12, and 27 months. The theoretical soil pH of each treatment, calculated by the liming rate model, was represented by corresponding dashed reference lines. The means ± standard deviation (*n* = 3) were shown. Statistical differences were denoted by lowercase letters using Tukey’s test at a significance level of *p* < 0.05.

Limestone application significantly increased the pH of smelting-affected soil during a maize–wheat rotation in northern China (Figure 2). The soil pH remained relatively stable around its native value of 4.80 over the 27-month observation period in the control treatment, reflecting natural fluctuations. However, the application of limestone altered this natural trend, leading to varying degrees of pH increases depending on the limestone ratios used. Specifically, the application of 2.25 t ha^−1^ or 4.45 t ha^−1^ of limestone resulted in a dramatic increase in soil pH throughout the 27-month sampling period, highlighting the effectiveness of limestone in improving smelting-affected soil in northern China (Figure 2). In the case of the 7.5 t ha^−1^ limestone treatment, soil pH showed a significant increase after 3–12 months, followed by a slight decrease after 27 months, potentially indicating the depletion of limestone (Figure 2).

### 3.2. Soil Cd Speciation

**Figure 3:**
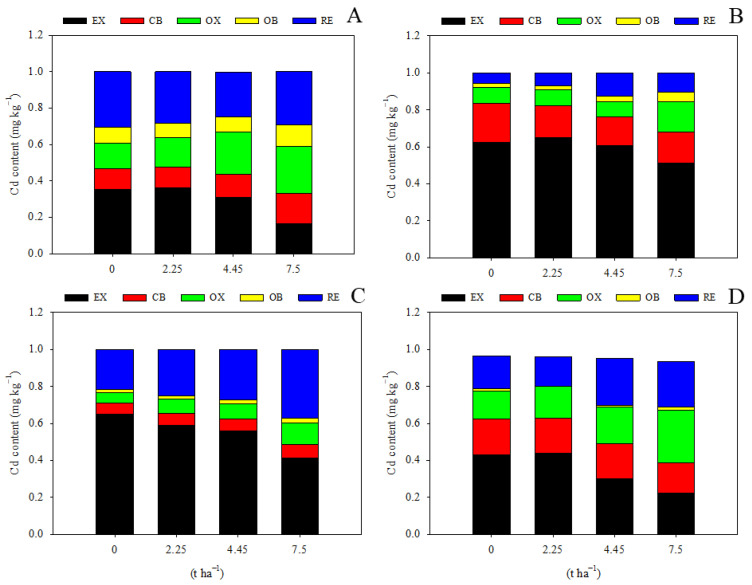
Limestone addition promotes soil Cd transformation significantly. EX, water-soluble and exchangeable Cd; CB, carbonate-binding Cd; OX, Fe/Mn-oxide-binding Cd; OB, organic-binding Cd; RE, residual Cd. (**A**) 3 months after limestone addition; (**B**) 9 months after limestone addition; (**C**) 12 months after limestone addition; (**D**) 27 months after limestone addition.

Limestone application had a significant impact on the speciation of Cd in the soil. With the exception of the treatment of 2.25 t ha^−1^ during the maize season, all other treatments led to a significant decrease in EX-Cd and an increase in RE-Cd, facilitating the transformation of soil Cd into a less bioavailable form (Figure 3). Application of 7.5 t ha^−1^ limestone resulted in a 53.6% reduction in EX-Cd, an 82.3% increase in OX-Cd, and a 39.5% increase in RE-Cd during the maize harvest season compared to levels observed two years prior during the maize season (Figure 3A), demonstrating the effectiveness of limestone. Interestingly, the content of RE-Cd did not show a significant response to limestone addition during the first maize growing season (Figure 3A), suggesting that a longer period of time is required for limestone to promote the transformation of soil Cd, as supported by data from the wheat season and the second maize season (Figure 3B–D). Moreover, it was observed that EX-Cd levels were relatively higher during the wheat maturation stage compared to maize, possibly due to seasonal variations in environmental conditions. Additionally, the levels of OX-Cd and OC-Cd were not significantly affected by the addition of limestone (Figure 3).

### 3.3. DTPA-Extractable Cd

**Figure 4:**
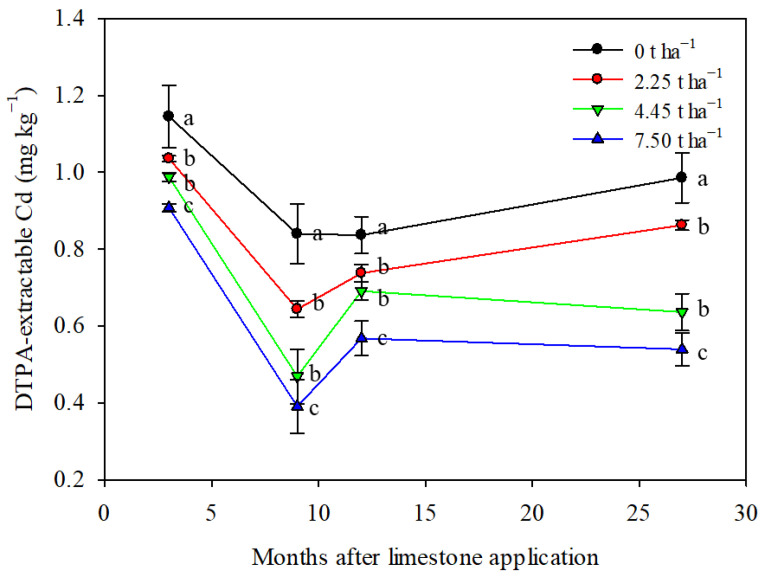
Limestone application decreased soil DTPA-extractable Cd content significantly during maize–wheat rotation in northern China. Means ± S.D. (*n* = 3). Statistical differences are inferred by lowercase letters by Tukey’s test, *p* < 0.05.

Limestone application had a significant impact on reducing the soil DTPA-extractable Cd content over a 27-month period (Figure 4). The control treatment showed fluctuations in soil DTPA-extractable Cd content, potentially influenced by seasonal variations or natural processes [42,43]. The addition of 4.45 and 7.50 t ha^−1^ of limestone notably changed the trend in soil DTPA-extractable Cd content compared to the control and 2.25 t ha^−1^ treatment. All limestone treatments effectively reduced the soil DTPA-extractable Cd content, demonstrating the long-lasting impact of limestone in controlling Cd bioavailability in the soil. The greatest reduction in soil DTPA-extractable Cd was observed with the application of 7.5 t ha^−1^ of limestone, resulting in a decrease of 20–54% after 3–27 months of limestone application (Figure 4). These results suggest that a single proper application of limestone can have a lasting effect for at least 27 months.

**Figure 5:**
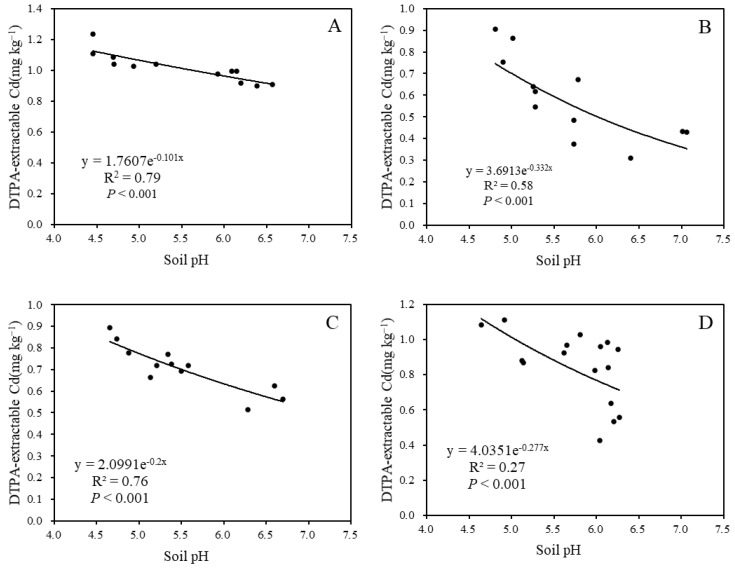
The relationship between pH and soil DTPA-extractable Cd: (**A**) after 3 months; (**B**) after 9 months; (**C**) after 12 months; and (**D**) after 27 months.

A significant negative correlation was observed between soil pH and DTPA-extractable Cd content after limestone application for 3 months, 9 months, and 12 months, with *R*^2^ values of 0.79, 0.58, and 0.76, respectively (Figure 5). This indicates a notable response of soil pH and DTPA-extractable Cd content to limestone usage.

### 3.4. Cd Content in Maize and Wheat

**Figure 6:**
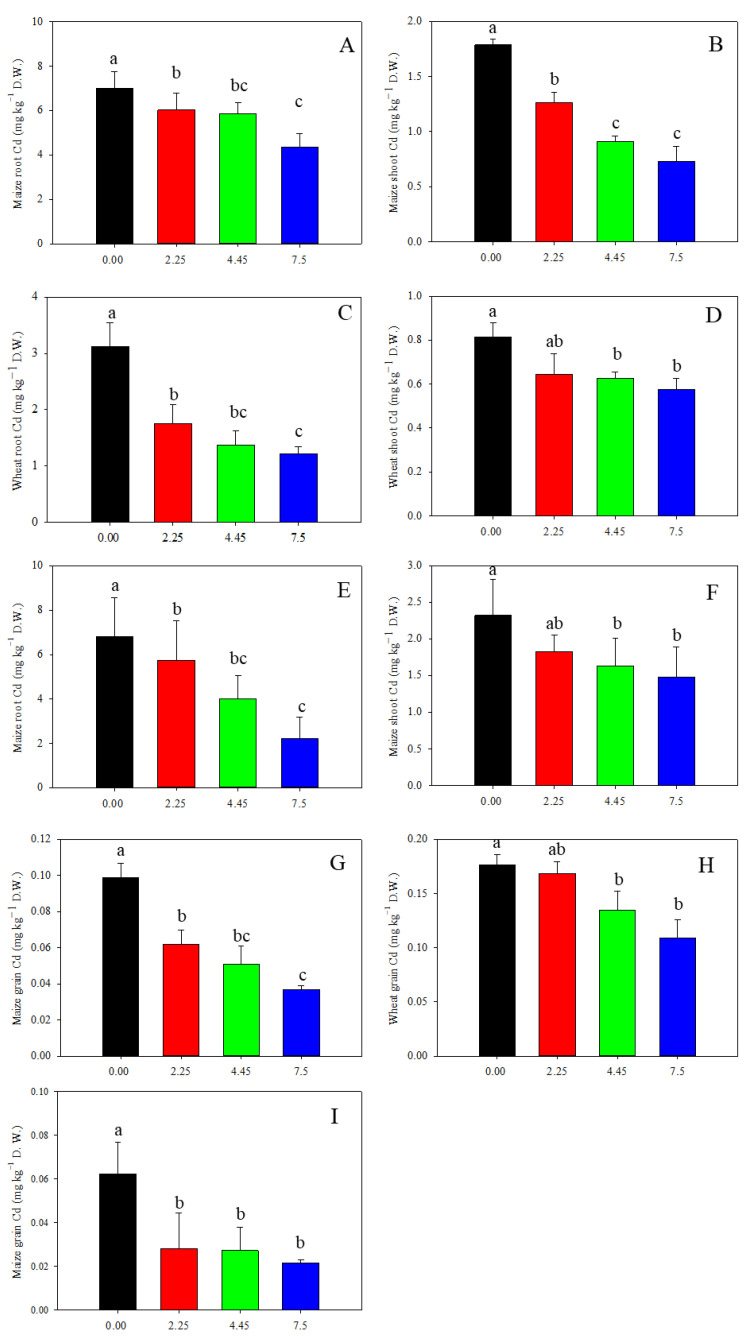
Limestone decreased wheat and maize Cd accumulation: (**A**,**B**,**G**) 3 months after limestone application; (**C**,**D**,**H**) 12 months after limestone application; and (**E**,**F**,**I**) 27 months after limestone application. Means ± S.D. (*n* = 3). D.W., dry weight. The lowercases indicate statistical significance by Tukey’s test, *p* < 0.05.

Limestone application had a notable impact on reducing Cd concentration in wheat and maize plants (Figure 6). In 2020, different doses of limestone led to a decrease of 5%, 24%, and 38% in wheat grain Cd content (Figure 6H). Similarly, in 2019, the Cd content in maize grain decreased by 37%, 49%, and 63%, and in 2021, decreased by 21%, 30%, and 36% after 3, 12, and 27 months of limestone application, respectively (Figure 6G,I). Furthermore, the accumulation of Cd in wheat grain was found to be higher than in maize grain (Figure 5). Despite the wheat grain Cd content exceeding the national food limit at 0.18 mg kg^−1^ in the control treatment, limestone application notably reduced this level. The results indicate that limestone effectively reduced Cd uptake by maize and wheat plants, with effects lasting up to 27 months. Additionally, the Cd content in wheat and maize roots and shoots also showed significant decreases following limestone application (Figure 6A–F).

### 3.5. The Relationship between Grain Cd and DTPA-Extractable Cd

**Figure 7:**
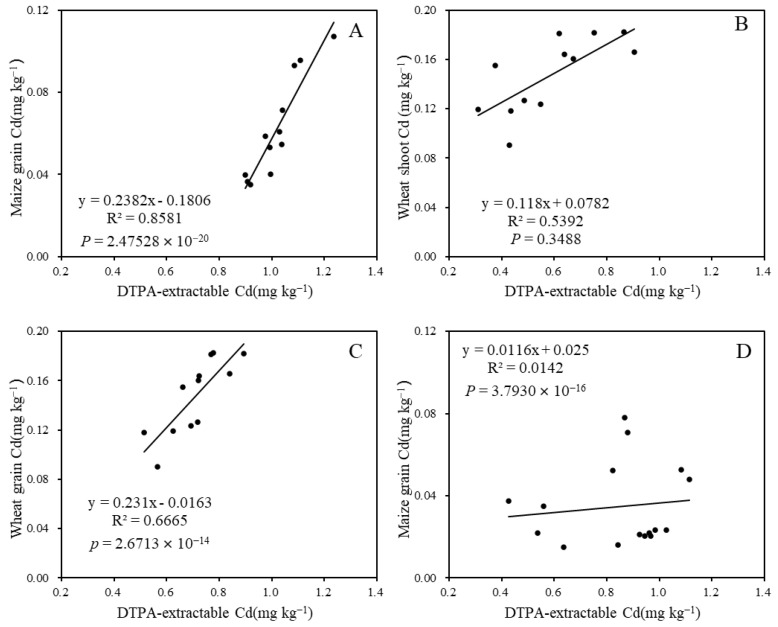
Correlation analysis between soil DTPA-extractable Cd and plant Cd content during various growth stages: (**A**) 3 months after limestone application; (**B**) 9 months after limestone application; (**C**) 12 months after limestone application; and (**D**) 27 months after limestone application.

Positive relationships between grain Cd content and soil DTPA-extractable Cd content were observed, with the highest correlation (*R*^2^ = 0.85) found in the 2019 maize maturation stage after 3 months of limestone application. The *R^2^* values for wheat shoot and grain Cd content in relation to their corresponding soil DTPA-extractable Cd were 0.58 and 0.64, respectively (Figure 7B,C). This suggests that soil DTPA-extractable Cd content significantly influenced wheat shoot and grain Cd accumulation, and could be notably reduced by limestone treatment.

### 3.6. The Effect of Limestone on Wheat and Maize Grain Yield

**Figure 8:**
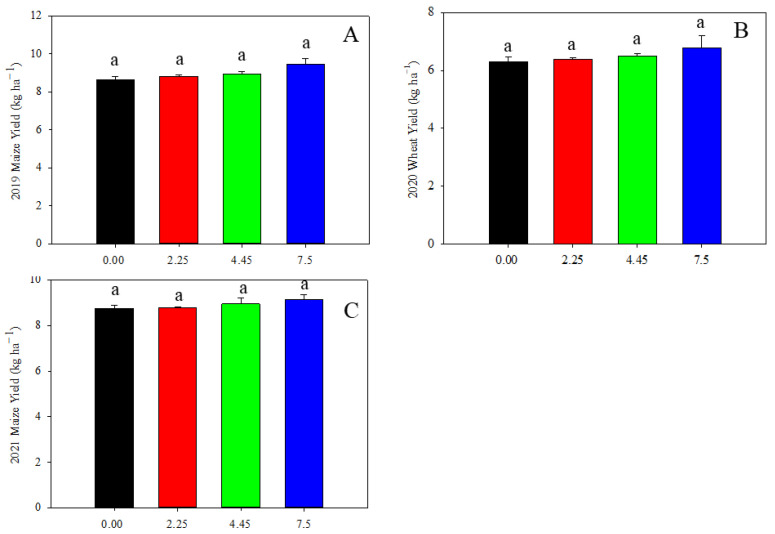
Cereal grain yield as affected by limestone addition: (**A**) maize yield in 2019; (**B**) wheat yield in 2020; and (**C**) maize yield in 2021. Data are shown as mean of three replicates ± S.D. Lowercase indicates significant statistical difference by Tukey’s test, *p* < 0.05.

We further charactered cereal yield as affected by limestone addition. The maize yield in 2019 and 2021, and the wheat yield in 2020 were not significantly affected by the addition of limestone (Figure 8).

## 4. Discussion

### 4.1. Limestone Addition Significantly Increased Soil pH

Limestone, a natural mineral found abundantly in the environment, is a cost-effective alternative to lime for remediating acid soils due to its milder action. While previous research has demonstrated the efficacy of limestone in improving acid paddy soils in southern China, its application in northern China and in maize–wheat rotations remains largely unexplored [36,44]. This study investigated the application of limestone to typical smelting-affected farmland in northern China. Utilizing a model developed from 23 acid soils in southern China, it was predicted that 7.5 t ha^−1^ of CaCO_3_ would raise the soil pH from 4.5 to 6.5 [36,37]. The findings of this study revealed a significant increase in soil pH in the smelting-affected farmland, indicating the effectiveness of limestone remediation in northern China (Figure 2). The observed improvements in soil pH aligned well with theoretical predictions, and the impact of 7.5 t ha^−1^ limestone application persisted for at least 27 months. These results are consistent with those from southern China, where 1.5 t ha^−1^ of lime raised paddy soil pH by an average of 0.50 units and a maximum of 1.40 units [44], while 7.5 t ha^−1^ of CaCO_3_ increased soil pH from 5.5 to 6.5 [36]. Also, the remediation effect is comparable with historical lime application in acid paddy soils in southern China, but without the concerns regarding its high causticity, quick reaction, and potential adverse effects on soil and plants [9,44].

### 4.2. Limestone Promotes Soil Cd Transformation and Decreases Cd Bioavailability in Smelting-Impacted Farmland in Northern China

Previous studies have shown that the addition of lime and other minerals to soil can lead to a similar redistribution of Cd. For example, lime addition reduced soil extractable Cd from 1.51 mg kg^−1^ to 0.70 mg kg^−1^ [45]. When lime was used as a soil conditioner, exchangeable Cd contents decreased significantly, while other Cd components increased [9]. Ca(OH)_2_ addition decreased exchangeable Cd components but increased inorganic bound Cd components [46]. In our study, we observed a decrease in exchangeable Cd components with increasing limestone dosage, along with an increase in other Cd components (Figure 3). This suggests that limestone is effective in transforming soil Cd into less bioavailable fractions, reducing plant uptake. Limestone raises soil pH, immobilizes heavy metals through solid-phase adsorption, and converts soluble metals to less bioavailable forms, thus decreasing Cd bioavailability [47]. Composite biomaterials containing CaCO_3_ have a strong affinity for Cd, forming precipitates and reducing Cd mobility in soil [48]. Biogenic CaCO_3_ has a high adsorption capacity for Cd, with adsorption being endothermic and spontaneous, following pseudo-second-order kinetics, ultimately reducing soil Cd bioavailability [49]. In our study, the soil DTPA-extractable Cd content significantly decreased with limestone addition, with the most pronounced decrease observed at 7.5 t ha^−1^ (Figure 4).

### 4.3. Limestone Decreases Wheat and Maize Grain Cd Accumulation

Compared to rice and maize, wheat exhibits more efficient Cd translocation to aboveground tissues, resulting in its overaccumulation in grains [19]. This phenomenon is largely attributed to the loss of function of the key gene *TaHMA3*, a close homolog of *OsHMA3*. In rice, *OsHMA3* primarily expresses in the roots, facilitating the transport of Cd into vacuoles and restricting its translocation to shoots [50]. The loss of function of *OsHMA3* leads to Cd overaccumulation in shoots and grains [38,50]. Wheat typically harbors loss-of-function alleles, but overexpression of *OsHMA3* from rice in wheat significantly reduces grain Cd accumulation [51]. Similarly, *ZmHMA3* plays a comparable role in Cd translocation in maize, with natural variation in *HMA3* alleles contributing to Cd accumulation differences in natural populations [52]. Despite this, maize grain generally contains lower Cd levels compared to wheat and rice [52]. This trend was observed in the current study, where Cd accumulation in maize grain in 2019 and 2021 was lower than that in wheat grain (Figure 6). This is further supported by frequent reports of Cd overaccumulation in wheat and rice grains, while instances of Cd content exceeding national food safety limits in maize grain are rare.

The study found that wheat grain Cd levels in the control treatment exceeded the national limit by 1.7–2 times, indicating a high phytoavailability of Cd in the soil tested, which had a DTPA-extractable Cd content of 0.79 mg kg^−1^. The pollution level was relatively low compared to northern China, but the soil’s low pH of 4.8 led to increased Cd availability to plants, resulting in excessive Cd accumulation in wheat grain. The application of limestone significantly reduced Cd uptake in maize and wheat grown on smelting-affected farmland in northern China. As the amount of limestone increased, Cd concentrations in the roots, shoots, and grains decreased. Specifically, 7.5 t ha^−1^ of limestone decreased the maize grain Cd content by 63% and 36% in 2019 and 2021, respectively, and reduced the wheat grain Cd content by 38% in 2020 (Figure 6). This reduction is similar to the remediation of Cd-contaminated paddy soil in southern China, where the addition of CaCO_3_ to high-yield rice cultivars decreased the rice grain Cd content by 70~80%, bringing it below the national food safety limit of 0.2 mg kg^−1^ [36]. In a study conducted in Hunan province, it was found that approximately 76% of rice grains exceeded the food safety limit for Cd content, but the application of 1.5 t ha^−1^ of burnt lime (75% CaO, <0.01 mm) increased the soil pH by 0.5 units and reduced rice grain Cd accumulation by 35.5% [44]. These results suggest that limestone is an effective method for reducing Cd accumulation in cereal grains, likely due to increased Cd fixation in the soil by raising the pH and reducing Cd bioavailability [53]. Additionally, it has been hypothesized that the addition of CaCO_3_ may competitively inhibit Cd uptake by Ca^2+^ plasma membrane transporters or channels in plants [54]. However, results from the addition of CaSO_4_ provide evidence against this hypothesis. A study found that iron minerals binding Cd became more prevalent after long-term flooding, and Cd accumulation decreased significantly with CaCO_3_ treatment compared to CaSO_4_ treatment in paddy soil [53]. This also indicates that the effect of limestone addition on soil pH is more important than competition sites in soil remediation.

The application of 7.5 t limestone per hectare significantly raised the soil pH and reduced the accumulation of Cd in wheat–maize grains, and this effect was observed to persist for at least 27 months (Figure 2, Figure 4 and Figure 6). This finding aligns with that of a previous study on rice paddy soils in southern China, where the remediation effect lasted for 2 years [36]. The 27-month duration of the remediation effect also suggests a practical approach for agricultural management, indicating that controlling soil Cd mobility can be achieved through limestone application every two years.

A strong linear relationship was observed between grain Cd content and soil DTPA-extractable Cd content, with the correlation between maize grain Cd content and soil DTPA-extractable Cd content being the most significant, indicated by an *R*^2^ value of 0.85 (Figure 7). This finding is consistent with similar results reported in other studies. Additionally, a negative correlation was found between soil EDTA-extractable Cd content and rice tissue Cd content with soil pH [47]. On the contrary, the *R*^2^ of 0.27 between soil pH and DTPA-extractable Cd content (Figure 5), and the *R*^2^ of 0.014 between DTPA-extractable Cd content and grain Cd content (Figure 7D) after 27 months of limestone application during the 2021 maize season may be attributed to the depletion of limestone in the 2.25 t ha^−1^ treatment or low accumulation of Cd in maize grain. The study suggests that the accumulation of Cd in wheat and maize grain can be predicted by calculating the amount of limestone added and limestone addition has no influence on grain yield (Figure 8). However, it is important to note that this research is limited to a specific area of smelting-affected farmland. Future studies should consider the remediation effect of limestone on the decline in soil pH resulting from nitrogen fertilizer application.

## 5. Conclusions

Limestone significantly increased soil pH, leading to a decrease in soil Cd bioavailability and Cd accumulation in wheat and maize grain. This effect persisted for at least 27 months in the 7.5 t ha^−1^ limestone treatment, demonstrating the feasibility of using limestone to control Cd accumulation in wheat and maize grain on soils impacted by the leaching of smelting tailing deposits.

## Data Availability

The raw data supporting the conclusions of this article will be made available by the authors on request.

## Supplementary Materials

The following supporting information can be downloaded at: https://www.mdpi.com/article/10.3390/toxics12080532/s1, Table S1: Basic properties of the soil in the present study.
