# Supplementary material for: Cadmium Minimization in Grains of Maize and Wheat Grown on Smelting-Impacted Land Ameliorated by Limestone
Source: Toxics. 2024 Jul 24;12(8):532. doi: 10.3390/toxics12080532 (PMC11359027; doi:10.3390/toxics12080532)
Supplement: Supplementary file 1 [file toxics-12-00532-s001.zip › toxics-3092025--supplementary.pdf]

Supplementary Files

Table S1 Basic properties of the soil in the present study

Available macroelements (mg kg^{-1})			Total metal (loid) content (mg kg^{-1})								DTPA-extractable metal(loid) content (mg kg^{-1})							
N	P	K	Cd	Cu	Mn	Fe	Zn	As	Pb	Cr	Cd	Fe	Mn	Cu	Zn	As	Pb	Cr
40	16	114	0.8	29	790	44000	147	17	116	70	0.6	121	63	6.8	5.3	0.98	20	8.5
